# Psychosocial, Behavioral, and HIV-Related Health Among Men Living with HIV Who Have a History of Incarceration

**DOI:** 10.1007/s10461-025-04887-5

**Published:** 2025-10-14

**Authors:** Chloe J. Kaminsky, Emily M. Cherenack, Sol Fernandez-Nocito, Jennifer Biondo, Yue Pan, Emily F. Dauria, Karen A. Johnson, Frank Palella, Matthew J. Mimiaga, Jennafer Kwait, Gina Wingood, Aruna Chandran, David B. Hanna, Catalina Ramirez, Morgan M. Philbin, Deborah L. Jones

**Affiliations:** 1 Department of Psychiatry and Behavioral Sciences, University of Miami Miller School of Medicine, Miami, FL USA; 2 Division of Pulmonary Medicine, University of Miami Miller School of Medicine, Miami, FL USA; 3 Department of Public Health Sciences, University of Miami Miller School of Medicine, Miami, FL USA; 4 Department of Behavioral and Community Health Sciences, University of Pittsburgh School of Public Health, Pittsburgh, PA USA; 5 The School of Social Work, University of Alabama, Tuscaloosa, AL USA; 6 Potocsnak Center for Aging and HIV, Northwestern University Feinberg School of Medicine, Evanston, IL USA; 7 Department of Epidemiology, Fielding School of Public Health, University of California Los Angeles, Los Angeles, CA USA; 8 Department of Psychiatry and Biobehavioral Sciences, David Geffen School of Medicine, University of California Los Angeles, Los Angeles, CA USA; 9 Institute for Health Research & Policy at Whitman-Walker, Washington, DC USA; 10 Mailman School of Public Health, Columbia University, New York, NY USA; 11 Johns Hopkins Bloomberg School of Public Health, Baltimore, MD USA; 12 Department of Epidemiology and Population Health, Albert Einstein College of Medicine, Bronx, NY USA; 13 University of North Carolina at Chapel Hill School of Medicine, Chapel Hill, NC USA; 14 Department of Medicine, Division of Health Equity and Society, University of California San Francisco, San Francisco, CA USA

**Keywords:** HIV, Incarceration, Depression, Substance use, Adherence

## Abstract

Incarceration is intricately linked with the HIV epidemic, with incarcerated individuals exhibiting HIV prevalence rates three times higher than the general population. We used baseline data from the Multicenter AIDS Cohort Study (MACS)/Women’s Interagency HIV Study (WIHS) Combined Cohort Study (MWCCS) to describe select health outcomes among men living with HIV (MWH) who have never been incarcerated and with a history of lifetime incarceration, specifically recent (within the past-year) and multiple incarcerations. Participants (*N* = 526) completed quantitative assessments of self-reported incarceration history, behavioral (e.g., antiretroviral therapy (ART) adherence, stimulant use) and psychosocial factors (e.g., depression, perceived stress), and provided biospecimens to assess immune function (i.e., CD4:CD8 ratio) and quantification of plasma HIV viral load. MWH with a history of any incarceration had significantly higher levels of depressive symptoms, recent stimulant use, detectable HIV viral load, and poorer ART adherence and lower CD4:CD8 ratios compared to MWH who had never been incarcerated. Among MWH with any incarceration, recent incarceration (versus not past-year) was associated with lower CD4:CD8 and poorer ART adherence. Recently incarcerated MWH were less often white and generally younger than MWH who had been incarcerated more than a year earlier, and MWH with multiple incarcerations more often identified as heterosexual compared to MWH who had only been incarcerated once. These findings underscore the critical need for tailored interventions aimed at MWH within a year following release from incarceration, focusing on enhancing mental health and HIV-related health outcomes.

## Resumen

El encarcelamiento está asociado con la epidemia de VIH. Usamos datos de referencia del Estudio de Cohorte Combinado del Estudio de Cohorte Multicéntrico de SIDA/Estudio Interinstitucional del VIH en Mujeres (MWCCS por sus siglas en inglés) para describir resultados en hombres que viven con VIH quienes nunca han estado encarcelados y quienes tienen un historial de encarcelamiento (reciente/en el último año, múltiples encarcelamientos). Participantes (*N* = 526) autorreportaron su historial de encarcelamiento, adherencia a su tratamiento antirretroviral (TAR), uso de estimulantes, depresión, estrés percibido, y proporcionaron especímenes biológicos por CD4:CD8 y carga viral. Hombres que viven con VIH con cualquier historial de encarcelamiento reportaron tasas elevadas de depresión, uso reciente de estimulantes, carga viral detectable, peor adherencia a su TAR y CD4:CD:8 más baja en comparación a hombres que viven con VIH quienes nunca han estado encarcelados. Encarcelamiento reciente (versus no en el último-año) fue asociado con CD4:CD8 más bajo, peor adherencia a su TAR, y demográficas más joven y menos blancos. Hombres que viven con VIH con múltiples encarcelamientos fueron más comúnmente heterosexuales en comparación con participantes que reportaron solo haber estado encarcelados una vez. Nuestros hallazgos resaltan una necesidad por intervenciones que mejoran resultados de salud mentales y relacionadas con el VIH en los hombres que viven con VIH quienes tienen historiales de encarcelamiento.

## Introduction

People who are incarcerated in the United States (US) are disproportionately impacted by HIV infection [1–3]. In 2021, 1.1% of individuals incarcerated in state and federal criminal legal institutions were living with HIV, making HIV prevalence among individuals who are currently incarcerated three times higher than among people in the general population [2]. Compared to people without HIV (PWoH), people living with HIV (PWH) have higher recidivism rates [4] and may be at an increased risk of adverse mental and physical health outcomes post-release [5]. Research is needed among men living with HIV (MWH) to understand the relationships between incarceration, mental health, health behaviors, and HIV outcomes and to identify if MWH with varied patterns of incarceration (e.g., recent or multiple incarcerations) are at elevated risk for adverse mental and physical health outcomes post-release [2].

Compared to PWH who were not recently incarcerated, PWH who have been incarcerated within the prior twelve months have higher odds of recently using drugs, receiving an HIV diagnosis in the past five years, having lower CD4 + T- lymphocyte cell (CD4) counts, and experiencing severe anxiety, and they are less likely to be virally suppressed [6]. Notably, PWH are more likely to have virally controlled HIV infection while incarcerated due to stabilization of care [7]; however, lack of access to care and subsequent decreases in antiretroviral (ART) adherence post-incarceration may contribute to higher HIV viral load and lower CD4 counts post-release [4].

In contrast, no mental health benefit of recent or multiple instances of incarceration have been reported [8]. In fact, lengthy stays in carceral facilities are associated with poorer mental health outcomes, such as depression and anxiety [9]. There is also a higher prevalence of mental health issues, such as depression, among PWH who have been reincarcerated (i.e., have been incarcerated multiple times) upon reentry into the community compared to persons who have not been reincarcerated, which may also contribute to substance use and poor ART adherence [10]. Additionally, previous studies have reported that declines in retention in care are more common in PWH who are reincarcerated than PWH who have not been reincarcerated [11], and cumulative incarceration events are associated with sexual risk-taking behaviors and sexually transmitted infections (STI) [12]. Another study identified cumulative burden of incarceration as a predictor of ART nonadherence among PWH, with the odds of ART nonadherence increasing as the number of incarceration events increased [13]. Therefore, PWH with both recent and multiple incidents of incarceration may be especially vulnerable to poor mental and physical health post-release; however, there is a need for additional research to characterize specific mental and physical health needs among recently and repeatedly incarcerated MWH.

Mass incarceration has significantly contributed to and sustained existing health disparities in the US, as the criminalization of mental illness and substance use disproportionately affects historically marginalized populations and people vulnerable for HIV [5]. Although both HIV and severe mental illness are overrepresented in the US criminal justice system, the association between multiple incarcerations and mental health among MWH has received minimal attention [14, 15]. Consequently, there are limited mental health resources tailored for MWH who have been recently or repeatedly incarcerated [16]. This study will address these gaps in the literature by characterizing mental health, health behaviors, and HIV clinical outcomes among MWH with recent and repeated incarcerations.

The current study used existing data from baseline visits collected in the nationwide Multicenter AIDS Cohort Study (MACS)/Women’s Interagency HIV Study (WIHS) Combined Cohort Study (MWCCS) to identify the prevalence of incarceration history, including lifetime incarceration, recent (within the past year) incarceration, and multiple incarcerations (being incarcerated more than one time) among MWH. Additional goals of the current study were to describe sociodemographic characteristics across incarceration histories and to evaluate the relationship between incarceration history and mental health, health behaviors, and HIV clinical outcomes among MWH. This cross-sectional analysis is intended to inform the design of future longitudinal and experimental studies.

## Methods

### Participants

This study used data collected from the MWCCS, an ongoing nationwide cohort study of PWH and PWoH [17]. Prior reports from the MWCCS have focused on women with HIV (WWH) who have been incarcerated, while the current study focuses on MWH [18, 19]. The MWCCS enrolls participants at sites across the US, including in Atlanta, GA, Miami, FL, Chicago, IL, Baltimore, MD, Los Angeles, CA, Birmingham, AL, Jackson, MI, Pittsburgh, PA, Columbus, OH, Bronx, NY, San Francisco, CA, Washington DC, and Chapel Hill, NC.

MWCCS inclusion criteria are being between the ages of 30 and 70 at enrollment, living with HIV or being at risk for HIV, and being willing to give written informed consent in either English or Spanish. To be eligible for the current analysis, MWCCS participants had to meet the following criteria: be a male between the ages of 30 and 70 and be living with HIV. The current study includes data collected at each participant’s first study visit from December 2020 to September 2023. All men newly enrolled in MWCCS were expected to have incarceration data available; men who were re-enrolled from the historical MACS study were not required to have completed the survey with incarceration variables but may have completed it based on local site protocols. Data were not collected during incarceration, so all instances of incarceration had occurred in the past. In total, 1188 MWH completed a study visit in the analysis time frame. Complete incarceration data was available from 92% (*n* = 498) of the 541 men newly enrolled in MWCCS who were expected to have incarceration data. Of 646 men re-enrolled from the historical MACS study, who were not required to have completed the incarceration form, 28 had complete incarceration data (4%). This resulted in a total of 526 men with complete incarceration data, with 8% missing data from newly enrolled participants who would be expected to have the incarceration variables completed.

### Ethical Considerations

All sites obtained approval from their institutional review boards, and all participants completed a written informed consent process prior to beginning any study procedures.

### Procedures

Substance use data and behavioral and social measures (i.e., depression and perceived stress) were collected via self-administered survey (ACASI), whereas all other variables were collected via interviewer-administered survey. Blood specimens were collected by trained study personnel for the measurement of HIV viral load (VL) and T-helper to T-suppressor ratio (CD4:CD8) using standard clinical measurement techniques at each site.

### Variables

#### Demographics

Demographic characteristics included age, race (i.e., American Indian/Alaskan Native, Asian, Native Hawaiian or Pacific Islander, Black, White, Other, Multi-Racial), ethnicity (i.e., Latino/Hispanic vs. Non-Latino/Hispanic), and sexual orientation.

#### Incarceration

Participants were asked whether they had been incarcerated (including in state or federal prisons, local jails, or other detention centers, such as immigration or overnight detention) in their lifetime (i.e., any lifetime incarceration), how many times they were incarcerated (i.e., incarceration frequency/being incarcerated more than once, which was operationalized as a binary yes/no variable), and if they had been incarcerated in the past year or not (i.e., incarceration recency, which was operationalized as a binary yes/no variable). Men who were incarcerated in the past year were asked if they took ART during their incarceration and if they were incarcerated in a state/federal or local facility for each instance of past-year incarceration.

#### Mental Health

Perceived stress was assessed using the Perceived Stress Scale (PSS-10), which results in a continuous outcome of 0 to 40, in which higher scores indicate higher perceived stress [20]. Depression was assessed via the Center for Epidemiologic Studies Depression Scale (CES-D), generating a score from 0 to 60, with a score of greater than or equal to 16 indicating elevated depressive symptoms [21]. Both the continuous depression score and a binary variable for elevated depressive symptoms were included in separate analyses.

#### Health Behaviors

We focused on stimulant use and ART adherence because both health behaviors have impacts on HIV progression and immune functioning among PWH [22]. Furthermore, stimulant use is one of the most used illicit substances among MWH [23]. We also considered examining opiate use; however, none of the participants reported opiate use in the past year. Participants self-reported use of any non-prescribed stimulants (e.g., methamphetamine, cocaine) in the past year. Participants who reported being prescribed oral ART by their provider were asked how often they took their ART in the past six months, with options being 100% of the time (perfect, or optimal, ART adherence), 95–99% of the time, 75–94% of the time, and less than 75% of the time.

#### HIV Clinical Variables

HIV VL was measured using the Aptima HIV-1 Quant Dx, COBAS 6800/8800 system, or the COBAS AmpliPrep assay, depending on site-specific protocols. Depending on site-specific thresholds, HIV VL was coded as undetectable when below 20–40 copies/mL. CD4:CD8 was measured using flow cytometry. For CD4:CD8, a ratio greater than one typically indicates optimal T-lymphocyte composition [24].

### Statistical Analyses

We examined sociodemographic variables (age, race, ethnicity, sexual orientation), mental health variables (perceived stress as a continuous score; depression as a continuous score; elevated versus normal depression), behavioral health variables (any versus no stimulant use in the last year; categorical ART adherence), and HIV-related variables (detectable versus undetectable HIV VL; CD4:CD8) across incarceration history. The current study conducted the following contrasts: (a) none versus any lifetime incarceration, (b) none versus one versus multiple incarcerations, (c) none versus recent (past year) versus distant (not in the past year) incarcerations. The purpose of these analyses was to identify any group differences based on incarceration history. Follow-up analyses further examined the differences between (a) multiple versus one incarceration and (b) recent versus distant incarceration by excluding men who had never been incarcerated from analysis; these follow-up analyses allowed us to test if there are unique characteristics among MWH recently or repeatedly incarcerated in comparison to MWH with just one incarceration or a distant incarceration history, which is critical for tailoring interventions to MWH with recent/repeated incarcerations. Fisher’s exact tests were used to assess the association of incarceration categories with categorical variables; analysis of variance (ANOVA) was used to compare means for continuous variables, except in the case where violations to ANOVA were identified, in which case the non-parametric alternative Kruskal-Wallis test was used. Lastly, a sensitivity analysis was conducted to assess if findings changed when limiting the sample to only newly enrolled MWCCS participants.

Alpha was set to *p* < 0.05. Analyses were conducted with R version 4.4.0 [25]. Missing data were generally minimal and excluded pairwise (Table 1). The sample size was pre-determined by the larger study.

**Table 1:** Demographic factors and key variables for men with HIV completing baseline assessments from December 2020 to September 2023

Baseline Characteristic	Total (*N* = 526)
Age	
Mean (± SD)	48.3 (± 11.4)
Median [Min, Max]	48.0 [30.0, 72.0]
Race, *n* (%)	
American Indian/Alaskan Native	1 (0.2)
Asian	4 (0.8)
Native Hawaiian or Pacific Islander	2 (0.4)
Black	301 (58.1)
White	145 (28.0)
Other	40 (7.7)
Multi-Racial	25 (4.8)
Ethnicity, *n* (%)	
Not Hispanic	421 (80.3)
Hispanic	103 (19.7)
Sexual Orientation, *n* (%)	
Straight	129 (25.1)
Gay/Queer	287 (55.8)
Bisexual/Pansexual	86 (16.7)
Other	12 (2.3)
* Missing*	12 (2.3)
Perceived Stress (PSS-10)	
Mean (± SD)	13.8 (7.13)
Median [Min, Max]	14.0 [0, 36.0]
* Missing*	24 (4.6)
Depression (CES-D)	
Mean (± SD)	12.3 (11.2)
Median [Min, Max]	9.00 [0, 54.0]
* Missing*	57 (10.8)
Elevated Depression (CES-D ≥ 16), *n* (%)	156 (32.4)
* Missing*	53 (10.1)
Stimulant Use in the Last Year, *n* (%)	62 (11.8)
Detectable HIV Viral Load, *n* (%)	126 (28.2)
* Missing*	79 (15.0)
CD4:CD8	
Mean (± SD)	0.969 (0.582)
Median [Min, Max]	0.900 [0, 3.30]
* Missing*	64 (12.2)
ART Adherence, *n* (%)	
100%	280 (57.3)
95–99%	155 (31.7)
75–94% 28	38 (7.8)
1–75%	14 (2.9)
0%	2 (0.4)
* Missing/No Oral ART*	37 (7.0)
Any Lifetime Incarceration, *n* (%)	186 (35.4)
Incarceration Recency, *n* (%)	
Never Incarcerated	340 (64.6)
Incarcerated > 1 year ago	171 (32.5)
Incarcerated < 1 year ago	15 (2.9)
Incarceration Frequency, *n* (%)	
Never Incarcerated	143 (27.2)
Incarcerated Multiple Times	43 (8.2)
Incarcerated One Time	340 (64.6)

Missing data not included in calculated percentages

## Results

### Participants

A total of 526 MWH had completed an assessment in the study timeframe and were eligible for analyses and included in the final sample (Table 1). The mean age was 48 years (Range: 30–72; *SD* = 11), and most participants were Black (58%), non-Hispanic (80%), and identified as gay or queer (56%). Regarding mental health, the mean perceived stress score among all participants was 14 (*SD* = 7), 32% of participants reported elevated depressive symptoms (*M*_CES−D score_ = 12, *SD* = 11), and 12% used stimulants in the past year. Most participants had an undetectable HIV VL (72%). Mean CD4:CD8 was 0.97 (*SD* = 0.58), and 93% reported taking oral ART, with 57% reporting perfect (i.e., 100%) ART adherence in the past six months. Two participants who were prescribed ART had not taken their medication at all in the past six months.

### Incarceration History

Overall, 35% of MWH were incarcerated at least once in their life, 3% were incarcerated recently (i.e., in the past year), and 27% were incarcerated multiple times. Of the 15 men incarcerated in the past year, there were 25 instances of incarceration, with men reporting a range from 1 to 4 incarcerations in the past year. Out of these 25 instances, ART was taken during incarceration at 60% of instances, 28% were at a state or federal prison, and 72% were in a local prison. Among the five men who did not take ART during incarceration (three with one instance of incarceration and two with two instances of incarceration), HIV VL was detectable in three individuals, undetectable in one, and not measured in one. ART adherence was 95–99% for the participants whose HIV VL was undetectable, and ART adherence was either below 95% (*n* = 3) or not measured (*n* = 1) in participants whose HIV VL was detectable. All participants who did not take ART while incarcerated were incarcerated in local facilities.

### Any Lifetime Incarceration Versus None

In an overall comparison of MWH who were never incarcerated versus MWH incarcerated at least once, statistically significant differences emerged by race, ethnicity, sexual orientation, depression, stimulant use, ART adherence, and HIV clinical outcomes (Table 2). No statistically significant association was found between any lifetime incarceration and age (*F*(1, 524) = 0.122, *p* = 0.727) or perceived stress (*F*(1, 500) = 2.871, *p* = 0.091). Compared to MWH who were never incarcerated, MWH incarcerated at least once were less likely to be White (14% among incarcerated vs. 36% among not incarcerated, *p* < 0.001) and more likely to be non-Hispanic (88% among incarcerated vs. 76% among not incarcerated, *p* = 0.004) and straight/heterosexual (49% among incarcerated vs. 12% among not incarcerated, *p* < 0.001). Compared to MWH who were never incarcerated, MWH incarcerated at least once had higher rates of elevated depressive symptoms (29% vs. 39%, *p* = 0.019), had higher mean depression scores (*M* = 12 vs. *M* = 14, *F*(1, 467) = 3.771, *p* = 0.053), more frequently used stimulants (8% vs. 19%, *p* < 0.001), had lower mean CD4:CD8 ratio (*M* = 1.01 vs. *M* = 0.89, *F*(1, 460) = 5.178, *p* = 0.023), more often had a detectable HIV VL (*p* = 0.016), and less frequently had perfect ART adherence (62% vs. 49%, *p* = 0.002).

**Table 2:** Sociodemographic characteristics, mental health, health Behaviors, and HIV clinical outcomes across any versus no lifetime incarceration among men with HIV

	Never Incarcerated (*n* = 340)	Incarcerated (*n* = 186)	F,^1^ *P*-Value
	*n* (%) or *M* (± *SD*)		
Age	48.1 (11.6)	48.5 (11.1)	0.122, 0.727
Race			
American Indian/Alaskan Native	1 (0.3)	0 (0)	< 0.001***
Asian	4 (1.2)	0 (0)	
Native Hawaiian or Pacific Islander	2 (0.6)	0 (0)	
Black	163 (48.8)	138 (75.0)	
White	120 (35.9)	25 (13.6)	
Other	32 (9.6)	8 (4.3)	
Multi-Racial	12 (3.6)	13 (7.1)	
Hispanic Ethnicity	81 (23.9)	22 (11.9)	0.004**
Sexual Orientation			
Straight	38 (11.6)	91 (48.9)	< 0.001***
Gay/Queer	226 (68.9)	61 (32.8)	
Bisexual/Pansexual	55 (16.8)	31 (16.7)	
Other	9 (2.7)	3 (1.6)	
Perceived Stress (PSS-10)	13.4 (7.03)	14.5 (7.28)	2.871, 0.091
Depression (CES-D)	11.6 (10.9)	13.7 (11.6)	3.771, 0.053
Elevated Depression (CES-D ≥ 16)	88 (28.6)	68 (39.3)	0.019*
Stimulant Use in the Last Year	26 (7.6)	36 (19.4)	< 0.001***
Detectable HIV Viral Load	69 (24.2)	57 (35.2)	0.016*
CD4:CD8	1.01 (0.587)	0.887 (0.565)	5.178, 0.023*
ART Adherence			
100%	195 (61.9)	85 (48.9)	0.002**
95–99%	95 (30.2)	60 (34.5)	
75–94%	21 (6.7)	17 (9.8)	
1–75%	3 (1.0)	11 (6.3)	
0%	1 (0.3)	1 (0.6)	

*p < 0.05. ** *p* < 0.01. ***p < 0.001

^1^F-Test (Anova) included for continuous variables

### Incarceration Frequency

When comparing variables across incarceration frequency (never vs. once vs. multiple), there were statistically significant differences by categories of race (*p* < 0.001), ethnicity (*p* = 0.013), sexual orientation (*p* < 0.001), elevated depressive symptoms (*p* = 0.034), stimulant use (*p* < 0.001), HIV VL (*p* = 0.046), CD4:CD8 (*F*(2, 459) = 4.362, *p* = 0.013), and ART adherence (*p* = 0.003) (Table 3). There were no significant differences for age (*F*(2, 523) = 0.241, *p* = 0.786), perceived stress (*F*(2, 499) = 1.486, *p* = 0.227), or depression measured as a continuous score (*F*(2, 466) = 1.905, *p* = 0.15). The group of MWH incarcerated multiple times had the lowest proportion of men who were White and Hispanic (11% White, 12% Hispanic), followed by MWH incarcerated once (23% White, 12% Hispanic), and never (36% White, 24% Hispanic). Sexual orientation differed across groups, with heterosexual being most common among MWH incarcerated multiple times (55%), followed by once (28%), followed by never (12%). Having elevated depressive symptoms was most common among MWH incarcerated multiple times (41%), followed by once (34%), followed by never (29%). A similar pattern emerged for stimulant use in the prior year, which was most frequently reported by MWH incarcerated multiple times (21%), followed by once (14%), followed by never (8%). Detectable HIV VL was least common for MWH never incarcerated (24%), compared to MWH incarcerated once (36%) or multiple times (35%). Mean CD4:CD8 was lowest among MWH incarcerated multiple times (*M* = 0.84), but similar for MWH incarcerated once (*M* = 1.04) and never (*M* = 1.01). Perfect ART adherence was most common among MWH never incarcerated (62%) and least common among MWH incarcerated multiple times (46%).

**Table 3:** Sociodemographic characteristics, mental health, health behaviors, and HIV clinical outcomes across incarceration frequency (multiple incarcerations, one incarceration, or no incarcerations) among men with HIV

	Never Incarcerated *(n =* 340)	Incarcerated One Time (*n* = 43)	Incarcerated Multiple Times (*n* = 143)	F,^1^ *P*-Value (3-Group Comparison)	*F*,^*1*^ *P*-Value (Multiple vs. One, *n* = 186)
	*n* (%) or *M* (± SD)				
Age	1 (0.3)	49.4 (± 11.4)	48.2 (± 11.0)	0.241, 0.786	0.383, 0.537
Race	4 (1.2)				
American Indian/Alaskan Native	2 (0.6)	0 (0)	0 (0)	< 0.001***	0.105
Asian	163 (48.8)	0 (0)	0 (0)		
Native Hawaiian or Pacific Islander	120 (35.9)	0 (0)	0 (0)		
Black	32 (9.6)	26 (60.5)	112 (79.4)		
White	12 (3.6)	10 (23.3)	15 (10.6)		
Other	81 (23.9)	3 (7.0)	5 (3.5)		
Multi-Racial		4 (9.3)	9 (6.4)		
Hispanic Ethnicity	38 (11.6)	5 (11.6)	17 (12.0)	0.013*	1.000
Sexual Orientation	226 (68.9)				
Straight	55 (16.8)	12 (27.9)	79 (55.2)	< 0.001***	0.005**
Gay/Queer	9 (2.7)	20 (46.5)	41 (28.7)		
Bisexual/Pansexual	13.4 (± 7.03)	9 (20.9)	22 (15.4)		
Other	11.6 (± 10.9)	2 (4.7)	1 (0.7)		
Perceived Stress (PSS-10)	88 (28.6)	14.8 (± 7.87)	14.4 (± 7.12)	1.486, 0.227	0.101, 0.751
Depression (CES-D)	26 (7.6)	13.3 (± 11.6)	13.8 (± 11.7)	1.905, 0.15	0.043, 0.837
Elevated Depression (CES-D ≥ 16)	69 (24.2)	14 (34.1)	54 (40.9)	0.034*	0.470
Stimulant Use in the Past Year	1.01 (± 0.587)	6 (14.0)	30 (21.0)	< 0.001***	0.382
Detectable HIV Viral Load		14 (35.9)	43 (35.0)	0.046*	1
CD4:CD8	195 (61.9)	1.04 (± 0.620)	0.840 (± 0.540)	4.362, 0.013*	3.742, 0.055
ART Adherence	95 (30.2)				
100%	21 (6.7)	23 (59.0)	62 (45.9)	0.003**	0.583
95–99%	3 (1.0)	10 (25.6)	50 (37.0)		
75–94%	1 (0.3)	3 (7.7)	14 (10.4)		
1–75%	1 (0.3)	3 (7.7)	8 (5.9)		
0%	4 (1.2)	0 (0)	1 (0.7)		

*p < 0.05. ** *p* < 0.01. ***p < 0.001

^1^F-Test (Anova) included for continuous variables

Follow-up analyses were conducted to compare characteristics among MWH who were incarcerated once versus MWH incarcerated multiple times while excluding MWH who were never incarcerated to investigate differences between multiple incarceration and a single incarceration. CD4:CD8 ratio was lower in MWH with multiple incarcerations (*M* = 0.84) compared to MWH with one incarceration (*M* = 1.04) (Table 3). Differences for CD4:CD8 were approaching significance (*F*(1, 164) = 3.742, *p* = 0.055). Sexual orientation also differed between groups, with a greater proportion of MWH in the multiple incarceration group identifying as heterosexual (55%) compared to the single incarceration group (28%), (*p* = 0.005).

### Incarceration Recency

When comparing MWH by incarceration recency (never vs. recent vs. distant), statistically significant differences emerged by categories for age (*F*(2, 523) = 8.042, *p* < 0.001), race (*p* < 0.001), ethnicity (*p* = 0.006), sexual orientation (*p* < 0.001), elevated depressive symptoms (*p* = 0.018), stimulant use (*p* < 0.001), HIV VL (*p* = 0.009), CD4:CD8 (*F*(2, 459) = 4.587, *p* = 0.011), and ART adherence (*p* = < 0.001) (Table 4). There was no statistically significant difference by categories of perceived stress (*F*(2, 499) = 2.367, *p* = 0.094) or depression as a continuous score (*F*(2, 466) = 2.55, *p* = 0.079). MWH with recent incarceration were younger on average (*M* = 37 years, *SD* = 6) compared to men with distant incarceration (*M* = 50 years, *SD* = 11) or no incarcerations (*M* = 48 years, *SD* = 12). MWH who were never incarcerated were more often White and Hispanic (36% White, 24% Hispanic) compared to MWH incarcerated distantly (14% White, 12% Hispanic) or recently (13% White, 13% Hispanic). Heterosexual orientation was most common among MWH distantly incarcerated (50%), followed by MWH recently incarcerated (33%), and never incarcerated (12%). Elevated depressive symptoms were most common among MWH recently incarcerated (57%), followed by distantly incarcerated (38%), followed by never incarcerated (29%). Similarly, stimulant use in the past year was most common among MWH recently incarcerated (27%), followed by distantly incarcerated (19%), followed by never incarcerated (8%). Detectable HIV VL was most common among MWH recently incarcerated (58%), followed by distantly incarcerated (33%), followed by never incarcerated (24%). CD4:CD8 was lowest among MWH recently incarcerated (*M* = 0.59), followed by distantly incarcerated (*M* = 0.91), followed by never incarcerated (*M* = 1.01). Perfect ART adherence was the least common among MWH recently incarcerated (14%), followed by distantly incarcerated (52%), followed by never incarcerated (62%).

**Table 4:** Sociodemographic characteristics, mental health, health Behaviors, and HIV clinical outcomes across incarceration recency (distant vs. recent (past-year) vs. no incarceration) among men with HIV

	Never Incarcerated *(n =* 340)	Distant Incarceration *(n =* 171)	Recent Incarceration *(n =* 15)	*P*-Value (3 Group Comparison)	*P*-Value (Distant vs. Recent, *n* = 186)
	*n* (%) or *M* (± SD)				
Age	48.1 (± 11.6)	49.5 (± 10.9)	37.3 (± 6.02)	8.042, < 0.001***	18.09, < 0.001***
Race					
American Indian/Alaskan Native	1 (0.3)	0 (0)	0 (0)	< 0.001***	0.557
Asian	4 (1.2)	0 (0)	0 (0)		
Native Hawaiian or Pacific Islander	2 (0.6)	0 (0)	0 (0)		
Black	163 (48.8)	128 (75.7)	10 (66.7)		
White	120 (35.9)	23 (13.6)	2 (13.3)		
Other	32 (9.6)	7 (4.1)	1 (6.7)		
Multi-Racial	12 (3.6)	11 (6.5)	2 (13.3)		
Hispanic Ethnicity	81 (23.9)	20 (11.8)	2 (13.3)	0.006**	0.695
Sexual Orientation					
Straight	38 (11.6)	86 (50.3)	5 (33.3)	< 0.001***	0.488
Gay/Queer	226 (68.9)	55 (32.2)	6 (40.0)		
Bisexual/Pansexual	55 (16.8)	27 (15.8)	4 (26.7)		
Other	9 (2.7)	3 (1.8)	0 (0)		
Perceived Stress (PSS-10)	13.4 (± 7.03)	14.3 (± 7.35)	17.0 (± 6.01)	2.376, 0.094	1.8, 0.181
Depression (CES-D)	11.6 (± 10.9)	13.4 (± 11.6)	17.1 (± 11.7)	2.55, 0.079	0.271, 0.271
Elevated Depression (CES-D ≥ 16)	88 (28.6)	60 (37.7)	8 (57.1)	0.018*	0.166
Stimulant Use in the Past Year	26 (7.6)	32 (18.7)	4 (26.7)	< 0.001***	0.495
Detectable HIV Viral Load	69 (24.2)	50 (33.3)	7 (58.3)	0.009**	0.115
CD4:CD8	1.01 (± 0.587)	0.914 (± 0.568)	0.593 (± 0.441)	4.587, 0.011*	4.224, 0.041*
ART Adherence					
100%	195 (61.9)	83 (51.9)	2 (14.3)	< 0.001***	0.002**
95–99%	95 (30.2)	55 (34.4)	5 (35.7)		
75–94%	21 (6.7)	14 (8.8)	3 (21.4)		
1–75%	3 (1.0)	8 (5.0)	3 (21.4)		
0%	1 (0.3)	0 (0)	1 (7.1)		

*p < 0.05. ** *p* < 0.01. ***p < 0.001

^1^F-Test (Anova) included for continuous variables

Follow-up analyses were conducted to compare MWH with a recent incarceration versus men with a distant incarceration while excluding MWH who were never incarcerated to compare MWH with a distant incarceration to MWH with a recent incarceration. Differences remained for CD4:CD8, which was lower in MWH with recent (*M* = 0.59) versus distant (*M* = 0.91) incarceration (*F*(1, 164) = 4.224, *p* = 0.041) (Table 4). Additionally, differences remained for ART adherence, with perfect ART adherence less common in MWH with recent (14%) versus distant (52%) incarceration (*p* = 0.002). MWH incarcerated recently were also younger on average than those who had a history of distant incarceration (*M*_*recent*_ = 37 vs. *M*_*distant*_ = 50, *F*(1, 184) = 18.09, *p* < 0.001).

### Sensitivity Analyses

Sensitivity analyses were performed to assess if findings changed when excluding men re-enrolled in MACS (versus newly enrolled in MWCCS), since men re-enrolled tended to be older on average. Results were virtually unchanged, with the exception that differences in HIV VL based on incarceration frequency (i.e., never vs. once vs. multiple times) were no longer statistically significant (*p* = 0.068), which may be due to a reduction in power.

## Discussion

This study examined sociodemographic characteristics, mental health, stimulant use and self-reported ART adherence and HIV clinical variables among MWH across different incarceration histories (i.e., incarceration recency and incarceration frequency groups). In the current study, all but one MWH who was recently incarcerated had also been incarcerated multiple times. This aligns with the US recidivism rates of approximately 70%, highlighting the need for reentry assistance and illuminating the difficulty of differentiating between the impact of recent versus repeated incarcerations, which frequently overlap [26, 27]. Our findings that Black, young, and heterosexual participants were more often recently and repeatedly incarcerated support well-established data on the disproportionate imprisonment of Black individuals in the US [28]. Findings suggest that interventions for MWH who have been recently and repeatedly incarcerated should be tailored to be relevant to Black, younger, and heterosexual MWH [29, 30]. This is critical, because many interventions for PWH and individuals at risk for HIV have been developed and tailored for sexual minority men [31].

MWH in this study with any history of incarceration were more likely to have elevated depressive symptoms, recent stimulant use, detectable HIV VL, worse ART adherence, and lower CD4:CD8 ratios compared to MWH who had never been incarcerated, which is aligned with prior research demonstrating associations between prior incarceration and adverse HIV clinical outcomes among MWH, such as elevated HIV VL, lower CD4:CD8, and non-optimal ART adherence [32, 33]. This study also builds on prior research suggesting individuals who were recently incarcerated are less likely to achieve viral suppression compared to those who were not recently incarcerated [34] by contributing novel insights on comparisons of multiple and recent incarcerations among MWH. In our study, MWH incarcerated multiple times had lower CD4:CD8 and were more frequently identified as heterosexual than MWH with one incarceration. MWH incarcerated recently were on average younger, had lower CD4:CD8 ratios, and were less likely to report perfect ART adherence than MWH incarcerated distantly. Among MWH recently incarcerated, about one-third were not taking ART. These participants who did not take ART were incarcerated in local facilities. Although the sample of MWH who were recently incarcerated and not taking ART was small, our finding that some men who were incarcerated in local facilities did not take ART is aligned with research demonstrating insufficient distribution of ART in jails and prisons relative to the known HIV infection rate in these settings [35]. In fact, qualitative research shows lack of access to ART as the greatest self-reported challenge in HIV care in the North Carolina Jail system [36].

Overall, this work suggests that MWH with any incarceration, especially MWH who have been incarcerated recently and multiple times, have the greatest risks for poor mental health, stimulant use, and suboptimal HIV clinical outcomes. Incarceration may put MWH at an additional risk of adverse HIV clinical outcomes due to associations between incarceration, substance use, and decreased ART adherence [19, 37]. Notably, it is also possible that MWH in this cohort who experience poor mental health and use stimulants will have more contact with the criminal legal system and consequent incarceration due to the criminalization of mental health and substance use disorders in the US [1]. Although previous research has also identified associations of incarceration with increased stress and anxiety among MWH, the current study did not find a significant difference between perceived stress among MWH with history of incarceration and MWH who were never incarcerated [6]. This could be a result of a lack of statistical power or indicative of a source of resilience among MWH, since coping can mitigate the impacts of a stressor on perceived stress, thus, improving immune function [38].

Our findings support the need for tailored interventions for MWH with a history of incarceration to reduce stimulant use and increase ART adherence and thereby improve virologic and immunologic outcomes, especially for MWH just released from criminal legal facilities. Some of the current health barriers after reentry for MWH include the lack of access to medical coverage, transportation, housing, and medical records from incarceration [39]. Intersectional stigma and discrimination experienced by MWH due to their HIV serostatus, carceral history, and race may contribute to difficulties in obtaining employment and housing, serving as a barrier to reentry and resulting in ongoing stigmatization post-release [40]. Successfully finding and maintaining housing may be especially difficult for MWH due to the high incidence of depression and substance use post-release, possibly due to unaddressed trauma while incarcerated [41]. Management of mental health challenges and improving coping skills may be essential pre- and post-release, which may also help reduce recidivism in this population [39, 41].

Similarly, substance use treatment during incarceration and post-release may be helpful for reentry. Although non-treatment programs such as drug education are most common in criminal legal settings, their efficacy is not clear; alternatively, some research suggests that medication-assisted treatment (MAT) and other longer, more intensive programs are more effective [42]. For example, providing medications for opioid use disorder reduces overdose deaths immediately upon release from the New York Jail System [43]; however, there is currently no Federal Drug Administration approved MAT for stimulants like methamphetamine and cocaine [44]. Research is needed to examine and develop the use of MAT for stimulant use among MWH who are currently incarcerated to lower the risk of substance use post-release.

In combination with substance use and mental health interventions, linkage to care is crucial for MWH who are incarcerated, as HIV care continuum outcomes appear to deteriorate among people once released from the carceral system [45]. Certain policies, such as Medicaid exclusion, may exacerbate this deterioration by preventing justice-involved populations from getting care during and post-release [46]. Receiving early linkage to care and receiving treatment and healthcare for HIV while in prison are associated with successful retention in HIV care post-release [10]. This emphasizes the need for policy reform and fast access to HIV care and ART medication in the carceral system, especially in local facilities, to ensure retention in care once released. Furthermore, time points during and post-incarceration may be critical in the HIV care continuum, and interventions undertaken while in criminal legal settings to improve HIV clinical outcomes post-release [47].

Ultimately, this work yields insight into adverse health outcomes that are more common among MWH who have been recently and/or repeatedly incarcerated and better characterizes the sociodemographic characteristics of MWH with repeated and recent incarcerations who are more vulnerable to adverse outcomes. This provides critical insights to better tailor interventions for young, Black, and heterosexual men with recent and repeated incarcerations to improve mental health, health behaviors, and HIV clinical outcomes.

Our findings should be interpreted considering the study’s limitations. This cross-sectional analysis should not be used to make causal inferences but can instead guide the design of future longitudinal studies that may consider the bidirectional effect of stimulant use on incarceration. For example, substance use and related circumstances, such as possession of drugs, can lead to incarceration due to the criminalization of substance use, mental health, and other behavioral risks [48]. However, incarceration may also lead to substance use, particularly the use of stimulants [49]. This is especially true of racial, ethnic, and sexual minority individuals who face a disproportionate risk of incarceration and substance use [50, 51]. It is important that future research considers the bidirectional relationships of stimulant use and incarceration and addresses structural racism and the consequent disproportionate mass incarceration of historically marginalized individuals as a contributor to public health crises and health disparities.

Follow-up analyses comparing groups within the incarceration frequency and recency categories, which exclude MWH who have never been incarcerated, were conducted to explore possible mental and physical health outcomes that may be uniquely associated with being recently or repeatedly incarcerated. However, these sub-comparisons, specifically within the recently incarcerated group, were made within a modest sample size. Furthermore, we made multiple theory-driven comparisons to assess essential aspects of health behaviors, mental health, and HIV clinical outcomes among MWH, but this could increase the likelihood of a multiple comparisons problem. Previous research has identified similar associations between recent incarceration and repeated incarceration and poor mental and HIV health outcomes [6, 34], increasing the likelihood that our results are generalizable and replicable; however, studies are needed to confirm the generalizability of findings across populations and determine the mechanisms linking incarceration history with mental health, health behaviors, and HIV clinical outcomes.

Future studies should compare the effects of incarceration between men and women to tailor interventions to meet the needs of both MWH and WWH who have been incarcerated. Research on WWH with a history of incarceration in the MWCCS has focused on associations between incarceration and increased sexual risk behavior and STI incidence, rather than mental health or HIV clinical outcomes; as a result, we cannot determine if the current study’s findings are generalizable to WWH in the US, and these outcomes should be investigated among WWH. Another avenue for future research should examine whether differences in health outcomes exist between types of detention (state/federal prisons, county jails, or other detention centers) and length of stay. Research suggests associations between incarceration and depression and substance use vary by type of facility, and further understanding incarceration contexts impacting MWH may help tailor interventions for different criminal legal settings [52]. Lastly, research is needed on broader mental health outcomes among PWH, such as post-traumatic stress disorder, which are prevalent among justice-involved populations and among PWH [53].

## Conclusion

MWH with a history of multiple incarcerations and those who have been incarcerated during the previous year may be at an elevated risk for suboptimal ART adherence, adverse HIV clinical outcomes, depression, and stimulant use compared to MWH who have never been incarcerated. Tailored reentry services and linkages to care interventions are needed for MWH both pre- and post-release. Future research should focus on interventions, policies, and public resources to mitigate the lasting impacts of incarceration on health among MWH.

## Declarations

### Research Involving Human and Animal Participants

All procedures performed in studies involving human participants were in accordance with the ethical standards of the institutional and/or national research committee and with the 1964 Helsinki declaration and its later amendments or comparable ethical standards. This study was approved by the IRB at all MWCCS sites.
